# Trends in dispensing of individual prescription opioid formulations, Canada 2005–2020

**DOI:** 10.1186/s40545-022-00423-1

**Published:** 2022-03-29

**Authors:** Wayne Jones, Ridhwana Kaoser, David Rudoler, Benedikt Fischer

**Affiliations:** 1 Centre for Applied Research in Mental Health and Addiction (CARMHA), Faculty of Health Sciences, Simon Fraser University, Suite 2400, 515 W. Hastings Street, Vancouver, BC V6B5K3 Canada; 2 Faculty of Health Sciences, University of Ontario Institute of Technology, 2000 Simcoe Street North, Oshawa, ON L1H 7K4 Canada; 3 Centre for Applied Research in Mental Health & Addiction, Faculty of Health Sciences, Simon Fraser University, 515 W. Hastings St., Vancouver, BC V6B5K3 Canada; 4 Department of Psychiatry, University of Toronto, 250 College Street, Toronto, ON Canada; 5 Department of Psychiatry, Federal University of São Paulo (UNIFESP), R. Sena Madureira, 1500, Vila Clementino, São Paulo, Brazil

**Keywords:** Canada, Dispensing, Formulations, Interventions, Opioids

## Abstract

**Background:**

Canada has experienced a distinctly bifurcated pattern of (strong) opioid utilization post-2000, with multifold increases rendering it one of the world’s highest opioid consumption rates, followed by subsequent substantive declines since 2011/2012. Several interventions to control especially high-risk opioid use have been implemented post-2010 at different levels, yet with their effects assessed mostly for overall opioid utilization. Little knowledge exists for over-time patterns of individual opioid formulations.

**Methods:**

Raw information on community-based prescription opioid dispensing for years 2005–2020 were obtained from a large national database based on a stratified sample of 6500 retail pharmacies across Canada (IQVIA/Compuscript), These data were converted into Defined-Daily-Doses/1000 population/day (DDD/1000/day) for individual (strong and weak) opioid formulations—specifically: fentanyl, hydromorphone, hydrocodone, morphine, oxycodone, codeine—per standard methods. Descriptive data on individual opioid dispensing were computed, and segmented regression (or ‘broken-stick’) analysis was applied to the overtime dispensing towards assessing potentially significant ‘breakpoints’ interrupting linear utilization trends. Akaike information criterion (AIC) values were computed to assess the resulting models’ quality-of-fit.

**Results:**

Five of the six opioid formulations featured a lower dispensing level in 2020 compared with 2005, but mostly with peak values in years between, contributing to the overall inversion pattern. For five of the six opioid formulations, a three-segmented model emerged as the best fit for the dispensing observed; only hydrocodone presented a linear (downward) dispensing trend. Among the five interrupted trend models for individual formulations, four (fentanyl, morphine, oxycodone, codeine but not hydromorphone) indicated their initial breakpoint during 2011–2014 introducing a downward dispensing trend. Inconsistently, morphine also featured a recent breakpoint (2018) towards a dispensing increase.

**Conclusions:**

While all opioids showed marked declines, we found heterogeneous patterns of dispensing for individual opioid formulations. While we cannot estimate direct causal effects, opioid control interventions appear to have had differential impacts on dispensing of individual formulations. The earliest breakpoint occurred towards substantive decreases for oxycodone dispensing in 2011; subsequently, there were increases in dispensing of hydromorphone and fentanyl likely due to substitution effects, followed by across-the-board declines post-2015/2016. Recent ‘safer opioid’ distribution programs to reduce illicit/toxic opioid exposure linked with high levels of poisoning fatalities seem to fuel resurgences in select opioid (e.g., morphine) dispensing.

## Background

Canada has featured a notable, while bifurcated pattern of medical opioid utilization post-2000. Canada’s total rate of controlled opioid dispensing (in Defined Daily Doses/million population/day; S-DDD] approximately tripled from 8713 S-DDD in 2000–2002 to 29,743 S-DDD in 2010–12, the then globally second-highest opioid consumption (after the United States [US]) [1]. These major expansions in opioid use were facilitated by multiple factors, including broad-based advocacy for improved treatment of ‘pain’, an expanding range of novel/potent opioid products, and aggressive marketing by pharmaceutical opioid producers [2–6]. However, the vast expansions in opioid availability have been accompanied by extensive adverse consequences, including major increases in the non-medical use, morbidity (e.g., opioid-related hospitalizations) and mortality (e.g., poisoning fatalities) related to opioids across Canada, gradually proliferating into an emerging ‘opioid crisis’. For example, in Ontario, Canada’s most populous province, opioid-related deaths and addiction treatment admissions increased by 55% and 60%, respectively, between 2004 and 2009 alone [7–10].

Few concrete policy interventions were initiated to reduce excess opioid availability until after 2010; in fact, a new, national ‘opioid guideline’ for chronic pain treatment released in 2010 yet presented recommendation for liberal and expanded opioid prescribing practices [11–13]. The initial intervention focus was on slow-release oxycodone (Oxycontin), which then represented about 20% of total opioid dispensing, but far larger proportions of population-level harm (e.g., non-medical use and fatalities) [7]. On this basis, most provinces removed slow-release oxycodone (‘Oxycontin’) from their public drug formularies (temporarily replacing it with a ‘tamper-proof’ version) in 2012, aiming to reduce hazardous opioid exposure in the population [14, 15]. This focus, however, neglected that multiple other strong opioids—for example, fentanyl and hydromorphone, representing approximately half of opioids consumed in 2012—were prevalently dispensed and contributing to opioid-related harm [1, 10, 16].

A range of other regulatory interventions were subsequently implemented on different levels [17]. Prescription monitoring program were strengthened, or newly introduced (e.g., Ontario in 2012) aiming to curb high-risk (opioid) use [18, 19]. In 2016, the US-based CDC’s new and restrictive opioid guideline was endorsed as a ‘clinical practice standard’ in select Canadian provinces (e.g., British Columbia) [20, 21]. A new national Canadian opioid guideline (2017) presented—paradigmatically revised—core recommendations for restrained, ‘last resort’-oriented opioid utilization in chronic pain care [22, 23]. In 2017, select provinces moved to delist high-strength opioid formulations from public drug plans [24]. Furthermore, the opioid crisis’ medical, economic, social aspects became a persistent focus of media investigations and reports during this period with likely impact on social attitudes, norms and practices [25, 26].

In these contexts, Canada’s opioid utilization rate peaked around 2012, and subsequently inverted and starkly declined (i.e., to 19,629 S-DDD in 2017–19) [1, 27]. Provincial opioid dispensing rates decreased by as much as 50% from their peak levels; half reported lower opioid utilization in 2020 compared with 2005 [28–30]. While various other interventions were implemented at different levels to restrain pharmaceutical opioid exposure, sudden while substantial increases in the availability of illicit, highly potent/toxic opioids (e.g., fentanyl) unfolded across Canada post-2015, vastly accelerating opioid-related harms [31]. For example, national annual opioid-related fatalities more than doubled from 2825 in 2016 to 6265 in 2020, mostly driven by increases in illicit opioid-related deaths [32].

Various analyses have assessed the impacts of interventions on changes in opioid utilization in Canada. For example, recent analyses identified significant downward ‘breakpoints’ in overtime strong opioid dispensing generally aligned with main interventions [28]. The implementation of the narcotics monitoring program and the delisting of high-strength opioid formulations in Ontario, and the introduction of the new opioid prescribing standards in BC were observed to result in reduced population-level opioid utilization or exposure [19, 24, 30]. Most pharmacoepidemiologic assessments of interventions—other than those specifically considering Oxycontin restrictions [15]—however have focused on total opioid utilization effects. Conversely, little knowledge exists on the patterns of the main individual opioid formulations in use. This paper presents a pharmacoepidemiologic examination of the dispensing of main individual opioid formulations in Canada 2005–2020, allowing for contextual assessments of how these individual groups of opioids may have been influenced by main opioid-related interventions implemented during this time.

## Methods

We obtained data on the community-based dispensing of prescription-based opioid formulations in Canada between 2005 and 2020 from the IQVIA Compuscript database. This commercial database collects and generates data from a representative and stratified sample of around 6500 retail pharmacies across Canada (representing approximately 60% of all retail pharmacies) [33] and is frequently used in pharmacoepidemiologic research [34–36]. The IQVIA database provides the yearly total number of prescriptions and units dispensed for each opioid by chemical formulation, product name, product form, and strength. While this database is estimated to cover approximately 80% of all prescription opioid dispensing in Canada, it does not include drugs dispensed in select other settings, such as hospital, internet or over-the-counter. We report opioid dispensing results for the six following main (strong and weak) opioids mainly utilized for chronic pain care in Canada: fentanyl, hydromorphone, oxycodone, morphine, hydrocodone, codeine (listed by generally accepted relative order of analgesic strength [37]). We excluded methadone from our analyses as methadone products are primarily used for opioid addiction treatment, and their application and dispensing differs between provinces and has been inconsistent overtime [16].

Using population estimates obtained from Statistics Canada [38] we converted the total national dispensing data for each of the opioid formulations examined into annual defined daily doses (DDD) per 1000 population per day (DDD/1000/day), based on the World Health Organization’s Anatomical Therapeutic Chemical classification [39]. DDD are a fixed unit of measure used to estimate the average dose of per day for a drug used in adults in a standardized manner, allowing for pharmacoepidemiologic comparisons primarily of quantitative use across different drug types and/or over time [39, 40].

Based on the measures described, segmented (or ‘broken-stick’) regression analyses were conducted on the annual dispensing values of each of the opioids between 2005 and 2020, in order to assess possibly abrupt (i.e., significant) shifts or changes in dispensing during this time. While some pattern changes can be visually identified based on observational data, the segmented regression allows to identify possible statistically significant interruptions or ‘breakpoints’ from linear trends [41]. The analysis partitions the independent variable into sub-intervals, while identifying possible inter-interval ‘breakpoints’, (e.g., with a two-segment model featuring one breakpoint, a three-segment model featuring two breakpoints, etc.).

The present analyses tested linear, two-segment, three-segment, and four-segment models for each opioid formulation’s dispensing pattern and determined the quality-of-fit of each model using the Akaike information criterion (AIC). We report results from the model that best fitted each opioid’s dispensing pattern as per the lowest (statistically significant) AIC value identified and the degree to which it was lower than the second-lowest AIC value. As a general rule-of-thumb, an AIC value less than or equal to two indicates little difference between the two models, differences between four and seven indicate some possible support for the second model, while differences greater than 10 indicate the first model is substantially better than the comparison [42]. All data manipulations and analyses were conducted using the R software [43] and its ‘segmented’ package [44].

## Results

**Fig. 1:**
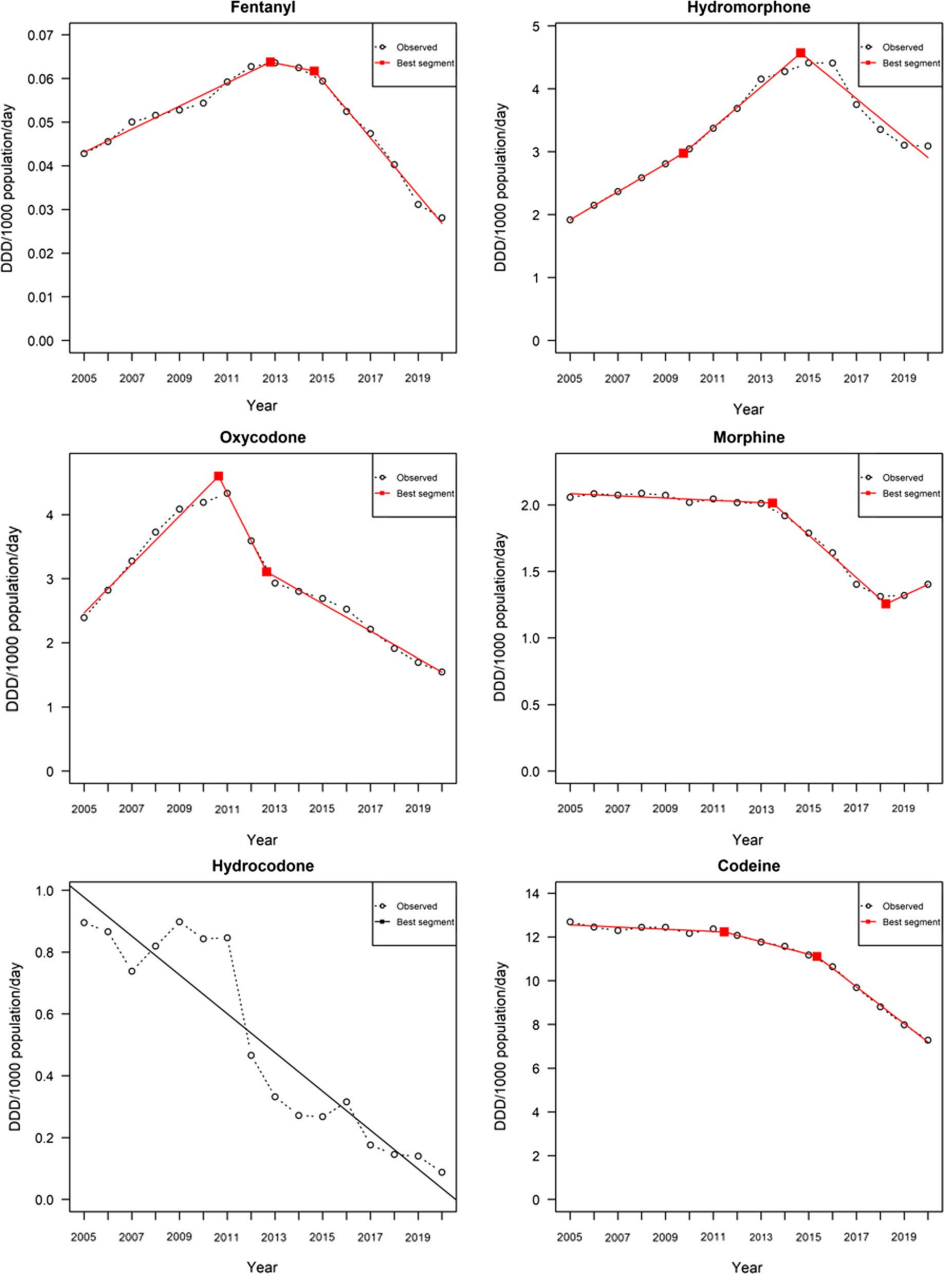
Plots of annual dispensing (2000–2020) of individual opioid formulations (in DDD/1000/day), including breakpoints, in Canada

Figure 1 visually presents the overtime dispensing trends, including breakpoints identified by the segmented analysis, in DDD/1000 population/day for each opioid from 2005 to 2020. The individual opioids are presented by generally accepted relative order of analgesic potency [37].

### Fentanyl

Dispensing of fentanyl rose from 0.04 DDD/1000/day to a high level of 0.06 DDD/1000/day (2011–2015) and subsequently declined substantially to a low point of 0.03 DDD/1000/day in 2020. A three-segment model emerged as the best fit, with the two breakpoints (initiating and accelerating declines, respectively) in dispensing identified in 2013 and 2015. The three-segment model had an AIC value of − 160.83 and adjusted R-square of 0.98 (*p*-value < 0.0001), presenting a slightly lower AIC value than the two-segment model AIC value: − 159.41).

### Hydrocodone

The dispensing pattern of hydrocodone—while with overall minor but non-significant fluctuations—overall decreased substantially, from 0.9 DDD/1000/day in 2005 to 0.09 DDD/1000/day in 2020. On this basis, the linear model, i.e., without any significant breakpoints identified, was the best fit with an AIC of − 18.34 and an adjusted R-square of 0.85 (*p*-value < 0.0001).

### Hydromorphone

The dispensing of hydromorphone more than doubled from 1.9 DDD/1000/day in 2005 to a high level of 4.4 DDD/1000/day in 2015/2016, and subsequently declined to 3.1 DDD/1000/day in 2020. A three-segment model was identified as the best fit, with breakpoints in 2010 (followed by an accelerating increase) and 2015 (inverting from increase to decrease). The three-segment model featured an AIC value of − 12.59 and adjusted R-square of 0.98 (*p*-value = 0.0004), yet only slightly lower than the two-segment model’s AIC value (− 12.45).

### Morphine

The dispensing of morphine held generally steady over the first ten years of the observation period (2.1–1.9 DDD/1000/day), after which it substantially decreased yet then slightly increased again to 1.4 DDD/1000/day in 2020. A three-segment model was the only model to attain statistical significance, including breakpoints identified in 2014 (initiating a downward trend) and 2018 (reverting to upward), and featuring an AIC value of − 64.40 and adjusted R-square of 0.99 (*p*-value = 0.03).

### Oxycodone

The dispensing levels of oxycodone almost doubled from 2.4 DDD/1000/day in 2005 to a high of 4.3 DDD/1,000/day in 2011, and subsequently decreased to an overall low of 1.5 DDD/1000/day in 2020. A three-segment model fitted the best, with breakpoints identified in 2011 (inverting from increase to decrease) and 2013 (decelerating decrease). The three-segment model featured an AIC value of − 20.80 and an adjusted R-square of 0.98 (*p*-value < 0.0001), lower than the two-segment model’s AIC value (− 1.38).

### Codeine

The dispensing of codeine showed an overall decreasing trend, declining by almost half from a high level of 12.7 DDD/1000/day in 2005 to a low level of 7.3 DDD/1000/day in 2020. A three-segment model emerged as the best fit, with breakpoints identified in 2012 and 2015 (both accelerating decreases). The three-segment model had an AIC value of -19.00 and an adjusted R-square of 0.99 (*p*-value = 0.03), with a lower AIC value than the two-segment model (AIC value: − 9.44).

## Discussion

Canada nationally has undergone a pronounced inversion pattern of medical opioid utilization, with substantial increases in opioid utilization in the first decade, and a reversal to similarly substantial declines in the context of multiple interventions implemented and aiming to reduce opioid consumption in the second decade of the period 2000–2020 [28, 36]. The markedly bifurcated evolution of opioid utilization during this period, however, has co-occurred with a proliferation of extensive opioid-related harms on the population level, including non-medical use, morbidity (e.g., hospitalizations and treatment-demand) and an unprecedented toll of opioid-related fatalities [45, 46]. While the general patterning, and the specific impacts of various individual interventions—mostly on overall opioid utilization—are documented, and some attention has been given to inter-provincial differences in opioid utilization, little comparative focus exists on the over-time patterning of individual opioid formulations [16, 19, 35].

Our examinations reveal both similarities, but also notable differences in dispensing patterns for main individual formulations, which may best be begun by considering ‘oxycodone’. In the early 2000s, the utilization of (slow-release) oxycodone products rapidly increased in Canada driven by several factors. The main brand product—Oxycontin—was aggressively (while misleadingly) marketed by its producers as an effective and safe medication for pain treatment, but emerged as a major driver in opioid-related harms (e.g., misuse, overdose deaths, etc.) following its introduction [7, 8]. Oxycontin became the initial core focus of the unfolding opioid crisis and emerging policy interventions, culminating in the de-scheduling of slow-release oxycodone from and its replacement with a ‘tamper-deterrent’ formulation on provincial drug formularies to reduce high-risk opioid use in 2012. This targeted intervention resulted in significant reductions—and related ‘breakpoints’ in dispensing identified—in oxycodone use in Canada starting at that time. More broadly, this specific intervention has been recognized as a turning point towards an increasingly restrained opioid-environment in Canada following years of liberal and extensive increases [14, 15].

The patterning of oxycodone was different from other main ‘strong opioid’ formulations, namely hydromorphone, fentanyl and—to some extent—morphine. These formulations made up more than half of Canada’s total opioid consumption by 2010, and were also associated with extensive adverse public health outcomes (e.g., overdose deaths) by the time the ‘opioid crisis’ gradually proliferated. To illustrate, they collectively accounted for 50% more opioid-related deaths than oxycodone (i.e., 337 and 229, respectively) in Ontario in 2011 [10]. Yet while restrictive formulary changes were implemented for oxycodone, hydromorphone and fentanyl both indicated continuously marked increases in dispensing, with only later initial breakpoints (2015 and 2013, respectively) towards inverting and declining consumption levels; correspondingly, morphine consumption remained at steady levels, and broke to a decline starting in 2014.

These inconsistent patterns are likely best explained by the fact that these strong opioid formulations—mainly hydromorphone—came to serve as substitutes formulations for the rapid reductions in oxycodone dispensing in contexts of persistent opioid demand and use [15, 47]. For example, national dispensing of oxycodone dispensing fell by 46.4%, but was partially offset by an increase of 47.8% in hydromorphone dispensing 2012–2016 [15]. In an overall context of increasingly restrained opioid utilization, latent decreases in these formulations’ use appear—temporarily—delayed as they were increasingly used to fill opioid prescription demand from existent patient populations following the forced reductions in oxycodone use. Each of them, however, begin to display reductions in use over the following years, contributing to the overall reductions in strong opioid use unfolding in Canada post-2012.

Notably, morphine and hydromorphone consumption indicate marked anomalies in consumption patterns more recently (2018 onward), in that their declining utilization trends broke and featured subsequent upwards or levelling trends. These reversals are likely best explained by novel interventions in response to recent massive increases in opioid-related fatalities mostly from illicit/toxic opioids (e.g., fentanyl and analogues) in Canada, which more than doubled, from 2825 in 2016 to 6214 in 2020 [29, 32, 48, 49]. Responding to these surges in accidental opioid mortality, several provinces have implemented growing numbers of local ‘safer opioid supply’ programs that distribute pharmaceutical grade opioids—mainly in the form of high-dose hydromorphone and slow-release morphine tablets—to at-risk opioid users as an overdose death prevention measure [50–52]. On this basis, Canada is undergoing recent resurgences in strong opioid dispensing not for expanding medications use in contexts of ordinary therapeutic (e.g., pain) care, but notably as an emergency-based public health measure responding to the—adverse while un-intended—consequences of marked oscillations in opioid availability and use.

Inconsistent dispensing patterns have been observed for the weaker opioids, i.e., hydrocodone and codeine, which followed a steep linear decline to virtual elimination, and a steady-to-decrease pattern, respectively. While these specific opioid types remained without specific, targeted interventions, the trends observed may be for several reasons. Hydrocodone products—which were commonly used in the US until tightening (e.g., up-scheduling) of controls occurred in 2014 [53]—were never used nearly as prevalently in Canada; in fact, their use was essentially restricted to the provinces of Ontario and Quebec. It is unclear for explaining the decline whether this drug simply fell out of favor by prescribers, there were ‘spillover’ effects from the US restrictions, or its use became gradually phased out in the larger ecology of increasingly restrained opioid use. Conversely, Canada’s codeine utilization rates—which traditionally have been high in global comparison based on both prescription and OTC use [54]—were steady but also began to decline post-2010, with further downward acceleration in 2015. Beyond wider ecological dynamics of opioid restrictions, these downward trends are likely driven by a cumulative set of positions and measures from medical stakeholder and regulators over the past decade that have generally questioned the efficacy and safety of, or directly advised against codeine use in certain risk groups (e.g., children, breastfeeding) all the way to calls for its complete de-licensing [55–57].

It appears evident that the different main opioid formulations examined have featured differential patterns in dispensing, but also appear to have responded differentially to the diverse array of—mostly restrictive—interventions aimed at medical opioid use implemented in Canada post-2010 [58]. This raises general questions about the overall coherence of the opioid policy and control approach implemented during this time. It is also noteworthy what the pharmacoepidemiologic data did not show evidence for certain possible change impacts. For example, the introduction of the new Canadian opioid guideline in 2017 did not correspond with breakpoints for significant trend changes for any of the main opioid formulations. This guideline represented a fundamental shift from previous—much more liberal and ‘pro-prescribing’—guidelines advising toward much more restrained (‘last resort’) opioid use chronic pain care [22, 59, 60]. Presumably, such an expert authority-based guidance tool would aid to discernably adjust or steer clinical practice for opioid utilization in discernable (here: decreasing) ways. In the US, the newly tabled CDC opioid prescribing guidelines (2016) were found to have resulted in significant decreases in opioid prescriptions both immediately before and following the guideline’s release [34]. Similarly, the CDC guideline’s implementation as a professional standard in BC (2016) was found to be associated with significant reductions in the number of opioid-receiving patients, both before and after its implementation [61]. Taken together, the data presented for Canada may suggest that these interventions may mainly reinforce, rather than independently impact on related behaviors (e.g., prescribing) influencing opioid dispensing patterns, and their messages may already be ‘factored into’ wider trends or dynamics when introduced; in addition, other factors or influences may drive or dominate related developments.

While it is commonly assumed that targeted policy or practice measures (e.g., ‘prescription guidelines’) centrally control medications use, other secular or ‘soft’ factors may influence related patterns in ways challenging for empirical definition and measurement. With regard to opioids, for example, there has been an extensive array of media investigations and reports in Canada mainly on the risks, harms and misguided practices related to opioid use post-2010 [26, 62, 63]. Plausibly, these contents may have influenced related medical-professional attitudes or practices, complementing or even pre-empting the impacts of more formal interventions.

The present study features some possible limitations. The community-based dispensing data used do not include all (but most) opioid dispensing which occurs in Canada. DDDs are a useful, while not consistently accurate measure for comparative opioid dispensing with regard to dosing [64]. Most opioid regulation parameters in Canada (total population: 38,037,000 [2020]) are province-based, while the provinces widely vary in population size (from 161,000 [Prince Edward Island] to 14,745,000 [Ontario]); thus, dispensing trends observed may be disproportionately driven by developments in the more populous provincial entities.

## Conclusions

Overall, Canada has undergone a pronounced evolution of an increase-to-decrease pattern in opioid dispensing through the period 2000–2020 [16, 28]. While each of the individual formulations underwent substantial declines at some point post-2011, this has involved rather differential dispensing patterns for the main formulations. To some extent, these differential patterns resemble the markedly heterogeneous pictures of opioid dispensing observed for the ten Canadian provinces, featuring rather wide ranges in consumption rates. While it was clear by 2010 that effective measures were required to reduce extensive opioid exposure and harms in the population, what followed was a somewhat disconnected mix of control interventions that affected individual formulations differentially. These impacts included lateral effects that increased opioid utilization, but also harms related to certain opioids seemingly substituting for reductions in others, associated with little or no total ‘net changes’ or even increases in key population health outcomes (e.g., opioid-related fatalities). Overall, while the majority of control interventions implemented have been assessed mostly for effects on total opioid use (by different metrics), pharmacoepidemiologic dynamics for individual formulations should routinely be evaluated for evidence-based policy evaluation and development.

## Data Availability

The raw data on prescription opioid dispensing in Canada were commercially obtained IQVIA Solutions Canada Inc., with all subsequent data processing and analyses solely conducted by the authors. Population data statistics used for the analyses were obtained from Statistics Canada. The external data sources/providers had no involvement or influence in any steps of the analysis development, results generation or data interpretation, or other aspects of the manuscript.

## Abbreviations

US: United States
DDD: Defined daily doses
CDC: Centers for disease control
AIC: Akaike information criterion
OTC: Over-the-counter
